# Hesperidin Inhibits the p53-MDMXInteraction-Induced Apoptosis of Non-Small-Cell Lung Cancer and Enhances the Antitumor Effect of Carboplatin

**DOI:** 10.1155/2022/5308577

**Published:** 2022-09-16

**Authors:** Yu Feng, Hongjie Huo, Qiong Tang

**Affiliations:** Department of Respiration Medicine, Tianjin Union Medical Center, Tianjin, China

## Abstract

**Objective:**

This study aimed to observe the effect of hesperidin on the apoptosis, proliferation, and invasion of non-small-cell lung cancer, as well as to explore the possible mechanism. The inhibitory effect of hesperidin combined with carboplatin on non-small-cell lung cancer was also investigated.

**Methods:**

A549 and NCI-H460 cells were treated with different concentrations of hesperidin (10, 50, and 100 *μ*M). The effect of siRNA knockdown on MDMX on the antitumor effect of hesperidin was observed. CCK-8 was used to detect cell activity. The apoptosis rate was determined by TUNEL. The transwell assay detects the ability of cell migration and invasion. The expression levels of the apoptosis-related proteins p53, MDM2, MDMX, p21, PUMA, Bcl-2, and Bax were detected by qRT-PCR. Cell-proliferation and transwell assays were used to detect the effects of the combined use of hesperidin and carboplatin on lung cancer cells.

**Results:**

Hesperidin significantly inhibited the activity and invasion of A549 and NCI-H460 cells in a dose-dependent manner. Hesperidin also induced the apoptosis of A549 and NCI-H460 cells. Hesperidin further inhibited the interaction between p53 and MDMX, increased the expression of p53, and played an anticancer role. The combination of hesperidin and carboplatin showed the most obvious antitumor effect.

**Conclusion:**

Hesperidin can inhibit lung cancer by inhibiting the interaction between p53 and MDMX. Moreover, the combination of hesperidin and carboplatin can inhibit the migration and invasion of lung cancer cell lines through p53 upregulation, thereby increasing the antitumor effect of carboplatin.

## 1. Introduction

Lung cancer is the most common and fatal respiratory malignancy in the world [1]. Non-small-cell lung cancer (NSCLC) accounts for more than 85% of the total number of lung cancer cases [2]. Most patients are in the middle and advanced stages by the time they are diagnosed and have thus lost the opportunity for surgery, so only 20% can receive surgical treatment [3]. Postoperative recurrence and metastasis rates remain as high as more than 50% [4]. Therefore, drug therapy has become the focus of NSCLC research [5–7].

The tumor-suppressor gene p53 plays an important role in the inhibition of tumor formation [8]. The murine double minute (MDM) family plays an important role in tumor genesis and development, especially in the regulation of p53 [9]. MDMX, also known as MDM4, can inhibit the transcriptional activation of p53 and regulate the E3 ligase activity of MDM2, and MDM2 and MDMX have nondependent tumorigenic effects of p53. Protein-protein interaction (PPI) is an important structural point in the process of tumor formation and development and is also one of the difficulties in targeted tumor therapy [10]. The development of PPI inhibitors for MDMX and P53 provides a new research direction for the treatment of NSCLC [11, 12].

Hesperidin is the main pharmacodynamic component of citrus fruits of *Rutaceae* [13]. Hesperidin has wide-ranging biological activities [14–16], including antioxidant, free-radical elimination, anticancer, and genotoxicity, as well as the ability to improve the bioavailability of other drugs. Hesperidin can induce tumor-cell apoptosis [17], inhibit tumor angiogenesis [17], and reduce tumor-cell proliferation [18]. The anti-inflammatory and antioxidant effects mediated by hesperidin are believed to be the main mechanisms of its antitumor effect [19], but the specific mechanisms have not yet been fully defined.

In recent years, platinum drugs including carboplatin have been extensively used in antitumor therapy [20]. These drugs are nonspecific anticancer drugs of the cell cycle [21]. Their efficacy is proportional to the dose, and their adverse reactions are also dose-related [22]. Accordingly, the dosage is often limited in clinical work due to the consideration of adverse reactions [23].

In the present study, the effect of hesperidin on the proliferation, apoptosis, and invasion ability of NSCLC cells was observed, and the mechanism of action was preliminarily discussed. We then combined hesperidin with carboplatin to provide an experimental basis for the development and application of hesperidin's antitumor activity. Overall, a new therapeutic strategy for the treatment of NSCLC was established.

## 2. Methods

### 2.1. Cell Culture and Transfection

Cells were cultured in a DMEM medium containing 10% fetal bovine serum and 1% streptomycin, and then placed in a 37°C incubator containing 5% CO_2_. Cell-growth density higher than 90% was considered for passage. Cells were resuspended regularly and the morphology of resuspended cells was observed. The growth curve was analyzed and pollution was detected. Follow-up experiments were carried out during the logarithmic-growth phase of cell culture. Hesperidin was divided into three doses with low (10 *μ*M), medium (50 *μ*M), and high (100 *μ*M) concentrations, and the treatment time was 24 h. Si-NC and si-MDMX were transfected using Lipofectamine™2000. The transfection method was based on the instructions of the Lipofectamine™2000 transfection reagent. The transfection efficiency was detected 48 h after transfection. Follow-up experiments were conducted 48 hours after transfection.

### 2.2. CCK 8 Experiments

A549 and NCI-H460 cells in a logarithmic-growth phase were collected and seeded onto 96-well plates. After incubation at 37°C and 5% CO_2_ for 12 h, the culture medium was discarded when the cells adhered to the bottom. The culture medium contained different concentrations of drugs and was added with 200 *μ*L. Culturing was continued until 72 h after dosing, with six compound wells set in each group. Then, CCK-8 10 *μ*L was added to each well for further incubation for 1 h. OD values of each well were measured by enzyme-linked immunoassay (BioTek) at 450 nm. The rate of cell proliferation-inhibition was calculated as follows: (control group OD–experimental group OD)/control group OD × 100%.

### 2.3. Transwell Experiment

The invasion ability of cancer cells was determined by transwell migration with the transwell coated by Matrigel (2.5 mg/ml). Cells are digested by trypsin and resuspended in a serum-free medium. The upper pores of the chamber were filled with cells from a serum-free medium (5 × 10^4^ cells/well) with hesperidin (50 *μ*M)/carboplatin (50 *μ*M). After incubation for 24 h, the noninvasive cells of the upper compartment were removed with a cotton swab and the cells on the lower surface were fixed. The cells were stained with 0.1% crystal violet solution for 15 min at room temperature for 15 min. Cells from four randomly selected fields were counted using a microscope. The experiment was repeated three times.

### 2.4. qRT-PCR

A549 and NCI-H460 cells at the logarithmic-growth stage were seeded onto six-well plates at 1 × 10^5^ cells/well. After the cells adhered to the wall, they were treated in groups according to the experiment. The cells were collected and the total RNA was extracted by TRIzol. RNA was reversely transcribed into cDNA using the Prime Script RT Reagent Kit. The samples were stored at −80°C for later use. cDNA was extracted for quantitative real-time PCR detection. The primer was synthesized by Shanghai Sangon Biological Engineering Company. Reaction conditions were as follows: 94°C for 2 min; 30 cycles at 92°C for 20 s, 56°C for 30 s, and 72°C for 45 s, with the last extension at 72°C for 7 min. The primers were as follows: Bcl-2, F: 5′-ATGCCTTTGTGGAACTATATGGC-3′, R: 5′-GGTATGCACCCAGAGTGATGC-3′; Bax, F: 5′-TGAAGACAGGGGCCTTTTTG-3′, R: 5′-AATTCGCCGGAGACACTCG-3′; MDMX, F: 5′-GCCTTGAGGAAGGATTGGTA-3′, R: 5′-TCGACAATCAGGGACATCAT-3′; P53, F: 5′-AGGGTTGGAAGTGTCTCATGC-3′, R: 5′-AAATCATCCATTGCTTGGGAC-3′; P21, F: 5′-AAGTCAGTTCCTTGTGGAGCC-3′, R: 5′-GGTTCTGACGGACATCCCCA-3′; PUMA, F: 5′-GACAGGAATCCACGGCTTTG-3′, R: 5′-TTCCATTCCGTTTCTTTTTCAGTT-3′; GAPDH, forward: 5′-GGGAGCCAAAAGGGTCAT-3′; reverse: 5′-GAGTCCTTCCACGATACCAA-3′. Detection results were calculated by the relative quantitative method of 2^−△△Ct^.

### 2.5. TUNEL

Cells were cultured in 6-well plates at a density of 3 × 10^5^ cells per well. Each group has 3 duplicate holes. After 24 hours of drug action, the medium was absorbed and discarded. It was washed 3 times with PBS. We refer to the operating instructions of the TUNEL staining kit (Beyotime, Shanghai, China). DAPI staining solution was added and stained for 5 min. The staining solution was removed and washed with PBS 3 times, 3 min each time. Then, we add an antifluorescence quenching sealing tablet to each slide. The cell apoptosis was observed under a laser confocal microscope (Nikon, Japan).

### 2.6. Statistical Analysis

SPSS17.0 software was used for statistical analysis. Results are expressed as the mean ± standard deviation. An one-way ANOVA was used for comparison between groups. *P* < 0.05 was considered statistically significant. Data counting was repeated three times.

## 3. Results

### 3.1. Hesperidin Induced Apoptosis and Inhibited the Proliferation and Invasion of NSCLC Cells

**Figure 1:**
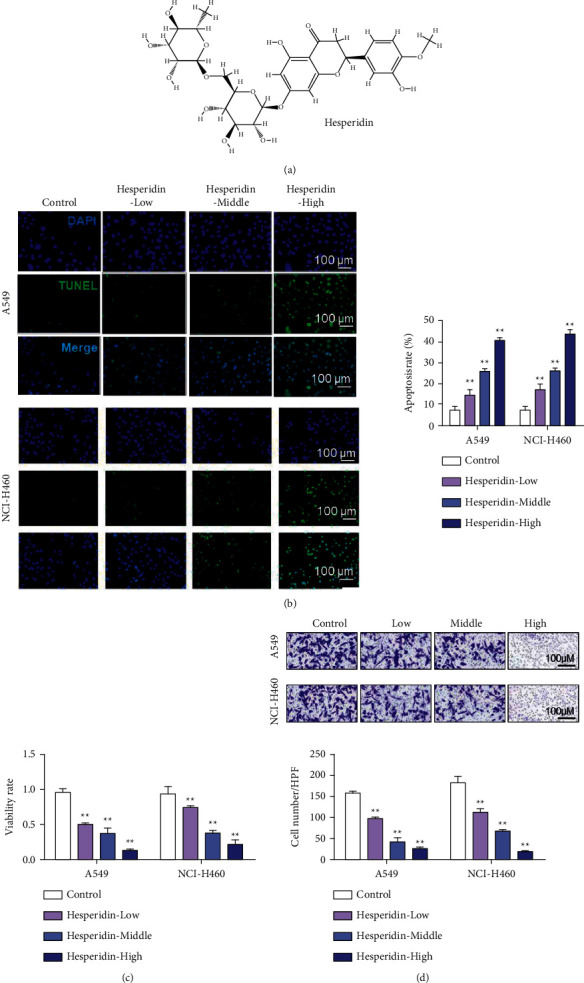
Hesperetin induced the apoptosis of wild-type p53 NSCLC cells and inhibited the growth of NSCLC cells. (a) Structural formula of hesperetin. (b) TUNEL to detect A549 and NCI-H460 cell apoptosis (hesperidin: 10, 25, and 50 *μ*M). (c) CCK-8 experiment to detect the changes in A549 and NCI-H460 cell viability. (d) Transwell test to detect the invasion ability of A549 and NCI-H460 cells. Error bars represent the standard deviations of three independent experiments. Scale bar = 100 *μ*m. ^*∗∗*^, *P* < 0.01. HPF: high-power field. Mean ± SD are shown, *n* = 3.

Hesperidin is a kind of flavonoid substance commonly found in citrus plants, and its molecular structure is shown in Figure 1(a). TUNEL was used to detect the apoptosis of A549 and NCI-H460 cells. The apoptosis rates of A549 and NCI-H460 cells treated with hesperidin for 24 h were significantly higher than those of the control group. Moreover, the apoptosis rate increased with the increase of hesperidin concentration in a dose-dependent manner (Figure 1(b)). Results suggested that hesperidin induced apoptosis in A549 and NCI-H460 cells in a dose-dependent manner. The CCK-8 assay was used to detect the changes in A549 and NCI-H460 cell activity. CCK-8 experimental results showed that A549 and NCI-H460 cells treated with hesperidin were significantly inhibited in a dose-dependent manner (Figure 1(c)). Compared with the control group, after treatment with different concentrations of hesperidin, the number of A549 and NCI-H460 cells invading and penetrating the membrane significantly decreased (Figure 1(d)). These findings suggest that hesperidin inhibited the invasion and migration of A549 and NCI-H460 cells and that the effect became more obvious with increased hesperidin dose.

### 3.2. Hesperidin Stabilized Wild-Type p53 by Destroying the p53-MDMX Interaction

**Figure 2:**
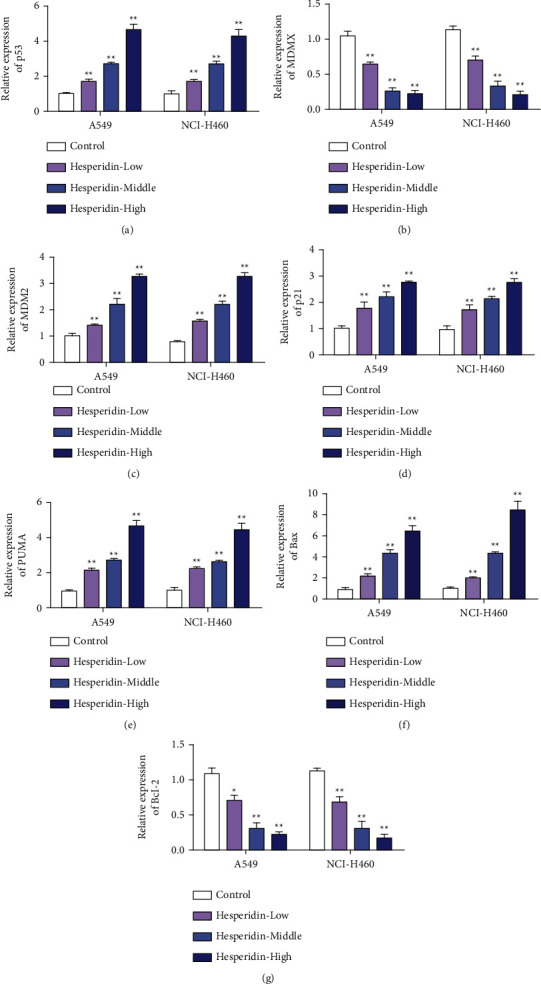
Hesperetin stabilized wild-type p53 by disrupting the p53-MDMX interaction. (a) Detection of the p53 expression after different treatments (hesperidin: 10, 25, and 50 *μ*M). (b) MDMX expression detection after different treatments. (c) MDM2 expression detection after different treatments. (d) Detection of p21 expression after different treatments. (e) PUMA expression detection after different treatments. (f) Bax expression detection after different treatments. (g) Bcl-2 expression detection after different treatments. Error bars represent the standard deviations of three independent experiments. ^*∗∗*^, *P* < 0.01. Mean ± SD are shown, *n* = 3.

To investigate the antitumor mechanism of hesperidin, we detected changes in the expression of p53-MDMX in A549 and NCI-H460 cells treated with different concentrations of hesperidin. Experimental results showed that hesperidin treatment could upregulate the expression of p53 in A549 and NCI-H460 cells (Figure 2(a)). However, hesperidin treatment inhibited MDMX expression levels in A549 and NCI-H460 cells in a concentration-dependent manner (Figure 2(c)). These results suggest that hesperidin increased the expression of free p53 by inhibiting the interaction of p53-MDMX. Furthermore, we detected changes in the p53 target expression levels of MDM2, p21, PUMA, and Bax in A549 and NCI-H460 cells. qRT-PCR results showed that hesperidin treatment upregulated the apoptosis-related genes MDM2, p21, PUMA, and Bax, with a significant dose-dependent relationship (Figures 2(c)–2(f)). The expression level of the antiapoptotic Bcl-2 was opposite to that of Bax. The expression of Bcl-2 mRNA in A549 and NCI-H460 cells was inhibited by hesperidin treatment and decreased with the gradual increase in hesperidin concentration (Figure 2(g)). These results suggest that the antitumor effect of hesperidin may be p53-dependent.

### 3.3. Hesperidin Induced p53-Dependent Apoptosis by Inhibiting the MDMX Expression

**Figure 3:**
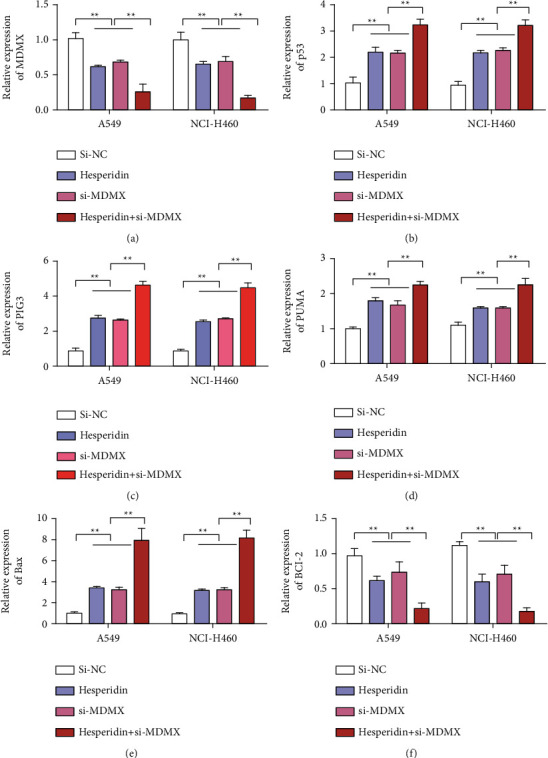
Hesperetin induced p53-dependent apoptosis by inhibiting the expression of MDMX. (a) MDMX expression detection after different treatments. (b) Detection of the p53 expression after different treatments. (c) PIG3 expression detection after different treatments. (d) PUMA expression detection after different treatments. (e) Bax expression detection after different treatments. (f) Bcl-2 expression detection after different treatments. Error bars represent the standard deviations of three independent experiments. ^*∗*^, *P* < 0.01; ^*∗∗*^, *P* < 0.01. Mean ± SD are shown, *n* = 3.

MDMX can regulate the function of p53 protein through interaction with p53 protein and posttranscriptional modification. After the siRNA knockdown of MDMX, we further investigated the role of MDMX in the antitumor action of hesperidin. First, the knockdown efficiency of si-MDMX was detected by qRT-PCR. Experimental results showed that siRNA inhibited the expression of MDMX in A549 and NCI-H460 cells (Figure 3(a)). Moreover, after adding hesperidin, MDMX expression also decreased. After transfection with si-MDMX and adding hesperidin, the MDMX expression level was the lowest (Figure 3(a)). The detection results of p53 expression showed that MDMX knockdown and hesperidin treatment upregulated the expression of p53. However, the p53 expression was the highest in the si-MDMX transfection group and hesperidin combined treatment group (Figure 3(b)). We also detected the expression levels of PIG3, PUMA, and Bax, which are related to apoptosis, and found that their expression levels were upregulated by hesperidin or si-MDMX treatment. PIG3, PUMA, and Bax had the highest expression levels in the si-MDMX transfection and hesperidin combined treatment group (Figures 3(c)–3(e)). Similarly, changes in the expression of the antiapoptotic Bcl-2 gene were also detected. The detection results of the Bcl-2 expression showed that hesperidin treatment and knockdown of MDMX inhibited the expression of Bcl-2 and that the combined group had the lowest expression (Figure 3(f)). These observations suggested that the mechanism by which hesperidin induced apoptosis was the activation of p53 through MDMX expression inhibition.

### 3.4. Nutlin-3a, an MDMX-Specific Inhibitor, Increased the Antitumor Effect of Hesperidin

**Figure 4:**
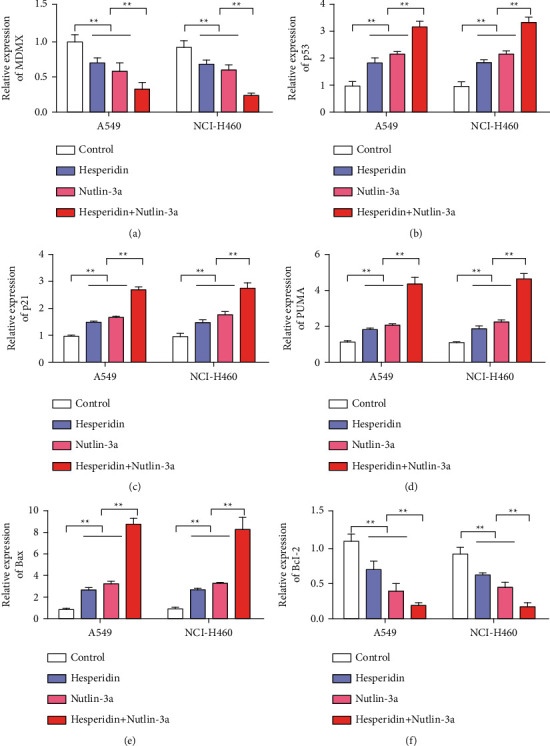
Hesperetin and Nutlin-3a (MDM inhibitor) synergistically inhibited p53-MDMX interaction. (a) MDMX expression detection after different treatments (Nutrin-3A: 10 *μ*M; hesperidin: 10 *μ*M). (b) Detection of the p53 expression after different treatments. (c) Detection of the p21 expression after different treatments. (d) PUMA expression detection after different treatments. (e) Bax expression detection after different treatments. (f) Bcl-2 expression detection after different treatments. Error bars represent the standard deviations of three independent experiments. ^*∗∗*^, *P* < 0.01. Mean ± SD are shown, *n* = 3.

The above experimental results indicate that MDMX inhibition can increase the antitumor effect of hesperidin. Accordingly, we further investigated the role of MDMX in the antitumor action of hesperidin after Nutlin-3a was used to inhibit MDMX. First, the inhibition of Nutlin-3a on MDMX was detected by qRT-PCR. Experimental results showed that Nutlin-3a inhibited the MDMX expression in A549 and NCI-H460 cells (Figure 4(a)). After adding hesperidin, MDMX expression also decreased. The MDMX expression was the lowest in Nutlin-3a combined with the hesperidin treatment group (Figure 4(a)). Results of p53 expression detection showed that Nutlin-3a or hesperidin treatment upregulated the expression of p53. The expression of p53 was the highest in the group treated with Nutlin-3a and hesperidin (Figure 4(b)). The expression levels of p21, PUMA, and Bax associated with apoptosis also showed that Nutlin-3a or hesperidin upregulated the expression of p21, PUMA, and Bax, which showed the highest expression in the group treated with Nutlin-3a and hesperidin (Figures 4(c)–4(e)). Changes in the expression of the antiapoptotic Bcl-2 gene were further detected. The detection results of Bcl-2 expression showed that Nutlin-3a or hesperidin treatment inhibited the expression of Bcl-2 and that the combined group had the lowest expression (Figure 4(f)).

### 3.5. Hesperidin Increased the Antitumor Activity of Carboplatin

**Figure 5:**
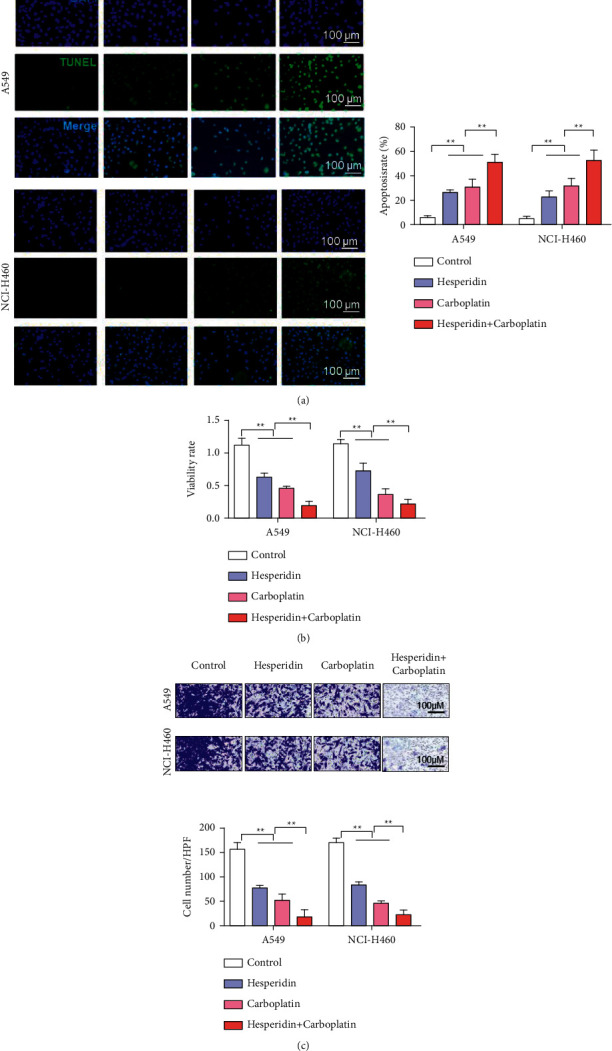
Hesperetin increased the antitumor activity of carboplatin. (a) Tunel to detect cell apoptosis (carboplatin: 40 *μ*M; hesperidin: 10 *μ*M). (b) CCK-8 experiment to detect cell proliferation after different treatments. (c) Transwell experiment to detect cell-invasion ability after different treatments. Error bars represent the standard deviations of three independent experiments. Scale bar = 100 *μ*m. ^*∗∗*^, *P* < 0.01. HPF: high-power field. Mean ± SD are shown, *n* = 3.

Carboplatin belongs to a nonspecific class of cell-cycle drugs and is a second-generation platinum antitumor drug. Carboplatin can produce interstrand and intrastrand crosslinks with DNA in tumor cells, resulting in DNA damage and leading to impaired DNA replication and transcription. It is often used in the clinical treatment of NSCLC. To further develop the clinical application value of hesperidin, we combined it with carboplatin and evaluated the combination's antitumor effect through cell-proliferation and transwell assays. Hesperidin- and carboplatin-induced apoptosis. A549 and NCI-H460 cells in the hesperidin + carboplatin treatment group had a synergistic effect with drugs and induced the highest apoptosis rate (Figure 5(a)). Results showed that hesperidin and carboplatin inhibited cell proliferation. The cell-proliferation rates of A549 and NCI-H460 were the lowest in the hesperidin + carboplatin treatment group. The combination exerted a synergistic effect on drugs (Figure 5(b)). Results of the cell-invasion analysis showed that hesperidin and carboplatin inhibited the invasion of A549 and NCI-H460 cells. The simultaneous treatment of hesperidin and carboplatin showed the most obvious inhibitory effect (Figure 5(c)).

### 3.6. Hesperidin Increased the Antitumor Effect of Carboplatin by Stabilizing p53

**Figure 6:**
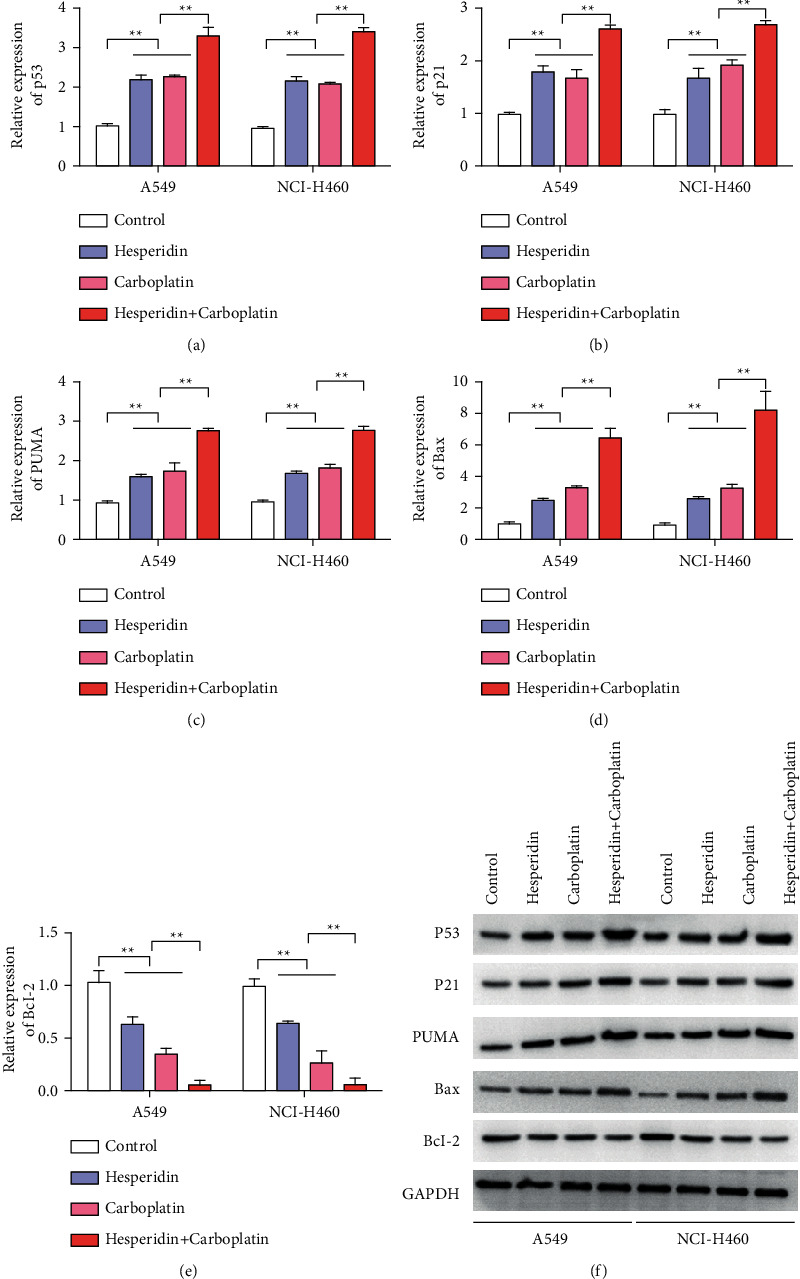
Hesperetin increased the antitumor activity of carboplatin by stabilizing p53. (a) Detection of the p53 expression after different treatments (carboplatin: 40 *μ*M; hesperidin: 10 *μ*M). (b) Detection of the p21 expression after different treatments. (c) PUMA expression detection after different treatments. (d) Bax expression detection after different treatments. (e) Bcl-2 expression detection after different treatments. Error bars represent the standard deviations of three independent experiments. ^*∗∗*^, *P* < 0.01. Mean ± SD are shown, *n* = 3.

First, we detected the regulatory effect of the combined application of hesperidin and carboplatin on p53 by qRT-PCR. Experimental results showed that hesperidin and carboplatin upregulated the expression of p53 in A549 and NCI-H460 cells (Figure 6(a)). The p53 expression level was the highest in the group treated with carboplatin and hesperidin (Figure 6(a)). The expression levels of apoptosis-related genes p21, PUMA, and Bax also showed that their expression levels were upregulated by carboplatin or hesperidin. In the group treated with carboplatin and hesperidin, the expression levels of p21, PUMA, and Bax were the highest (Figures 6(b)–6(d)). Similarly, changes in the expression of the antiapoptotic Bcl-2 gene were also detected. The detection results of the Bcl-2 expression showed that carboplatin or hesperidin treatment inhibited the expression of Bcl-2, and the combined group had the lowest expression (Figure 6(e)). These observations suggest that the mechanism by which hesperidin increased the antitumor effect of carboplatin was achieved by stabilizing p53.

## 4. Discussion

Lung cancer remains the most fatal malignancy [24]. Chest radiographs, low-dose CT, and other early screening methods are extensively used in the early detection of lung cancer [25]. However, most patients at the time of diagnosis are already in an advanced stage of the disease and thus lose the chance of surgical treatment. At present, the methods of medical treatment primarily include chemotherapy, molecular targeted therapy, and immunotherapy [26, 27]. The application of chemotherapy increases the 1-year survival rate of advanced NSCLC by 10%, which is the cornerstone of the treatment of advanced NSCLC [28]. Nevertheless, reducing the toxic and side effects of chemotherapy drugs and developing adjuvant chemotherapy drugs are highly significant [29–31].

Hesperetin, a dihydroflavone compound, is a secondary metabolite of various plants and is widely distributed in the plant kingdom [32]. Hesperidin can inhibit the growth of tumor cells in a time-dose-dependent manner [33–35]. Studies on the survival and apoptosis of chronic myeloid leukemia cells [36], as well as esophageal and breast-cancer cells [37], have shown the antitumor effects of hesperidin. In our experiments, hesperidin and carboplatin alone were found to inhibit the growth of A549 and NCI-H460 cells in a concentration-dependent manner. Combined use of hesperidin and carboplatin showed a significantly higher inhibition rate than single use, indicating a synergistic effect. Hesperidin can significantly reduce the dose of carboplatin on A549 and NCI-H460, promoting the efficacy of chemotherapy and reducing the toxic and side effects at the same time.

P53 inhibits tumor growth through various pathways, including cell-differentiation inhibition, apoptosis promotion, senescence, and autophagy [38]. Under stress signals, p53 can activate the transcriptional expression of downstream target genes and inhibit the occurrence of tumors [39]. Multiple proteins can bind to p53 to play the role of ubiquitin ligase, leading to p53 degradation, such as MDMX [38]. MDMX is usually highly expressed in tumors and has a long half-life [40]. The molecular mechanism of MDMX regulating p53 and the process affecting tumor development have not been fully elucidated [41]. Gembarska et al. [42] demonstrated that inhibiting the interaction between MDMX and p53 and restoring the function of p53 can increase the sensitivity of tumors to chemotherapy. Haupt et al. [43] suggests that MDMX induces breast cancer knockout and inhibits cell growth, and this effect is p53-dependent. Wang et al. [44] showed that the use of NS-C207895 in the breast-cancer cell line MCF-7 to inhibit the expression of MDMX can activate p53 and lead to the high expression of proapoptotic genes, thereby promoting the apoptosis of tumor cells. In the present study, hesperidin was found to be a small-molecule inhibitor capable of acting on the p53/MDMX system. Hesperidin inhibited the interaction among p53 protein, MDM2, and MDMX and thus enabled p53 to play a normal biological function and an antitumor role. Although hesperidin is not clinically available, it has the potential to become an activator of the p53 protein. Moreover, because hesperidin inhibited p53-MDMX interaction, it did not cause extensive stress stimulation to cells. From the perspective of precision therapy, hesperidin may be suitable for patients with NSCLC with high MDMX protein expression.

The tumor gene p53 plays an important role in the occurrence and development of NSCLC. As an effective transcription factor, p53 is activated in response to cellular stress and regulates a large number of downstream target genes. These genes are involved in cell cycle control, apoptosis, angiogenesis, and cell senescence. The antitumor effect of p53 protein is mainly to block the G1/G0 phase of the cell cycle so that cells cannot enter the S phase, thus inhibiting cell growth. At the same time, p53 protein can enter the nucleus, bind with specific DNA, promote gene expression, and play a role in transcriptional activation of cell suppressor genes. The p53-MDMX regulatory axis is more dominant in p53 [45]. It plays an important role in the normal physiological activities of cells, as well as the formation, progression, treatment, and prognosis of tumors [46]. In the present study, we found that hesperidin promoted the release of p53 and restored the independent function of p53, playing the role of a tumor-suppressor gene by inhibiting the interaction between p53 and MDMX. Finally, we found that hesperidin could be combined with carboplatin to increase the antitumor effect of carboplatin. The combination of hesperidin and carboplatin has advantages. First of all, the combination of drugs can exhibit a synergistic antitumor effect to improve efficacy. At the same time, drug combinations can delay or reduce the emergence of drug resistance. Combinations can also reduce individual drug doses, thereby reducing toxic side effects. The deficiency of this study lies in the need to further improve the understanding of this target and to apply the research results to the clinical settings as soon as possible, which is also an urgent problem to be solved in the future. In addition, other mechanisms of hesperidin inhibiting lung cancer cells should be further studied.

## 5. Conclusion

The effective rate of conventional chemotherapy for lung cancer is low and has some adverse reactions. This experiment confirmed that hesperidin can inhibit the proliferation and metastasis of NSCLC by inhibiting the interaction of p53 and MDMX. Hesperidin can also enhance the chemotherapy sensitivity of carboplatin and reduce the dosage of carboplatin. Hesperidin may pave a new way for the prevention and treatment of lung cancer by overcoming some adverse reactions of traditional carboplatin. However, the antitumor effect and mechanism of hesperidin require further study. With the research and development of hesperidin analogs and further study of their antitumor mechanisms, hesperidin may become a promising candidate compound for the prevention and treatment of tumors.

## Data Availability

The datasets used and/or analyzed in the current study are available from the corresponding author on reasonable request.

## Abbreviations

NSCLC: Non-small-cell lung cancer
MDM: Murine double minute
PPI: Protein–protein interaction
ANOVA: Analysis of variance.
